# Effects of oral gavage with periodontal pathogens and plaque biofilm on gut microbiota ecology and intestinal tissue architecture in mice: a mechanistic study

**DOI:** 10.3389/fcimb.2025.1589055

**Published:** 2025-08-08

**Authors:** Lan Huang, Song Ge, Kun Yang, Lian Duan, Li Gao, Yu Zhen Li, Yu Shi Yi

**Affiliations:** Periodontal Department, Affiliated Stomatological Hospital of Zunyi Medical University, Zunyi, China

**Keywords:** *Fusobacterium nucleatum*, *Porphyromonas gingivalis*, *Streptococcus sanguinis*, oral-gut axis, dysbiosis, tight junctions, 16S rRNA sequencing

## Abstract

**Objective:**

This study aimed to establish an *in vitro* model simulating periodontal biofilm architecture with three representative periodontal pathogens and evaluate its systemic impact through oral gavage administration in C57BL/6 mice. The findings provide mechanistic insights into the oral-gut axis dysbiosis, elucidating potential pathways linking periodontal inflammation to gastrointestinal pathophysiology.

**Methods:**

Fifty 7-week-old male C57BL/6 mice were randomized into five groups(n=10/group): control (H), *F. nucleatum* (F), *P.gingivalis* (P), *S.sanguinis* (S) and biofilm (BF, *F.n* + *P.g* + *S.s*) groups. Mice were gavaged twice weekly for 6 weeks with 1×10^9^ CFU (F, P, BF groups) and 1×10^8^ CFU (S group) of bacterial suspensions or PBS (H group). Post-intervention, fecal and colon tissues were collected for 16S rRNA sequencing, H&E staining, immunohistochemistry (Occludin expression), and qRT-PCR analysis of inflammatory markers(IL18, TNF-α, IL-1β, B220, F4/80, NOS2, ARG1).

**Results:**

A stable *in vitro* three-species biofilm model was successfully established to mimic the ecology of periodontal plaque. Gavage with *F.n*, *P.g* or the biofilm consortium (BF group) induced intestinal barrier disruption and elevated pro-inflammatory cytokines levels. PCR indicated a significant increase in the expression of IL-1β, TNF-α, B220, F4/80, and NOS2 in the P group (*P* < 0.001), while Arg-1 expression exhibited a significant decrease *(P* < 0.01). In the BF group, only TNF-α expression demonstrated a significant increase (*P* < 0.01). The expression of occludin is significantly reduced in the F/P/BF group, with the most pronounced decrease observed in the P group (*P <* 0.01). Gut microbiota alterations occurred in all groups. At the phylum level, the *Firmicutes/Bacteroidetes* (F/B) ratio increased in all three groups (F/P/BF group). *Proteobacteria* abundance rose substantially in the P group, while *Desulfovibrio* increased and *Verrucomicrobia* decreased in the F/P/BF and F/S groups, respectively. Genus-level analysis showed reduced *Muribaculaceae* in the F/P/BF group, alongside elevated pro-inflammatory bacteria (e.g., *Enterococcus*, *Acinetobacter*) and diminished beneficial bacteria (e.g., *Bifidobacterium*, *Parabacteroides*).

**Conclusion:**

These findings demonstrate that periodontal pathogens induce gut barrier compromise through microbiome-driven immunomodulation, with *P. gingivalis* exhibiting predominant pro-inflammatory effects.

## 1 Introduction

Periodontitis and inflammatory bowel disease (IBD) represent chronic inflammatory disorders driven by immune dysregulation and microbial dysbiosis. Emerging evidence underscores bidirectional interactions between these conditions, mediated through direct microbial translocation (e.g., oral pathobiont gut colonization) and indirect systemic immune priming (Caruso et al., 2020). Notably, periodontal therapy may attenuate systemic inflammation, while IBD management could reciprocally improve periodontal outcomes, highlighting the clinical imperative for dual-pathway interventions (Qing et al., 2024). As a globally prevalent chronic infection affecting >50% of Chinese adults (30% severe cases; Jiao et al., 2021), periodontitis arises from dysbiotic subgingival biofilms dominated by keystone pathogens. Socransky’s microbial complex theory delineates progression-associated consortia: the red complex (*Porphyromonas gingivalis, Tannerella forsythia, Treponema denticola*) and orange complex (*Fusobacterium nucleatum, Prevotella intermedia*) drive tissue destruction, while early colonizers like the yellow complex (*Streptococcus sanguinis, S.mitis*) facilitate biofilm maturation (Socransky et al., 1998).

*P.gingivalis*, a principal pathogenic bacterium, exhibits a multifaceted pathogenic mechanism. Its adhesion and aggregation factors facilitate colonization within periodontal pockets. Additionally, the lipopolysaccharides (LPS) it secretes can inhibit the expression of host chemokines, thereby evading the host’s immune defenses (Mao et al., 2023). Furthermore, toxic factors such as gingipains produced by this bacterium can directly induce tissue degradation and provoke excessive inflammatory responses. *F.nucleatum*, characterized by its extensive lectin system, not only mediates the co-aggregation of early and late colonizing bacteria as a “biological bridging molecule,” but also significantly prolongs the survival of *P.gingivalis* by inhibiting metronidazole activity, thereby creating a synergistic pathogenic effect (Sakanaka et al., 2022). As a core early colonizer, *S. sanguis* establishes the structural foundation for subsequent colonization by periodontal pathogens through the regulation of biofilm matrix formation (Lima et al., 2017). Dental plaque biofilms are typically classified into supragingival and subgingival categories. Subgingival biofilms (advancing front), particularly non-attached anaerobic flora, exhibit architectural resilience through extracellular polymeric substance(EPS)-mediated ecological optimization, conferring antimicrobial resistance and sustained pathogenicity (Jakubovics et al., 2021). These analyses reveal critical microbial parallels between periodontal and peri-implant ecosystems. While *Rothia*, *Neisseria*, and *Corynebacterium* constitute core commensals in healthy periodontal niches, these genera demonstrate inflammatory priming potential in peri-implant mucositis through pathogenicity state transitions. 16S rRNA gene amplicon sequencing identified disease-specific microbial signatures, with peri-implantitis exhibiting increased microbial diversity and pathogen-enriched community shifts compared to periodontitis, including both classical and emerging periodontal pathogens (Di Spirito et al., 2024). Notably, the microbial dysbiosis patterns in peri-implantitis show phylogenetic convergence with enteric disorders, suggesting a compelling rationale for investigating oral-gut axis interactions as a priority research direction.

The oral-gut axis (Tortora et al., 2023; Zhou et al., 2023) has emerged as a critical interface in systemic disease pathogenesis, including gastrointestinal disorders such as irritable bowel syndrome, IBD and colorectal cancer (CRC) (Wang and Fang, 2023; Zhu et al., 2024a), as well as Alzheimer’s disease (AD) (Qian et al., 2024), cardiovascular diseases (Priyamvara et al., 2020), and diabetes (Li et al., 2023). Gut equilibrium is upheld by a tripartite defense system: a physical barrier formed by tight junction proteins, a chemical defense provided by the mucus layer, and immune surveillance conducted by gut-associated lymphoid tissue (Zhao and Maynard, 2022). Periodontal pathobionts disrupt gut homeostasis through: (1) Mucin-mediated gastric acid resistance enabling gut colonization (Kato et al., 2018); (2) Tight junction degradation via MMP-9 activation (occludin/ZO-1 downregulation); (3) Th17-mediated chronic inflammation (Yao et al., 2022).

Clinical correlations include *F. nucleatum/P. gingivalis* enrichment in colorectal cancer microbiota (Pignatelli et al., 2021), while murine models demonstrate oral pathogen-induced gut dysbiosis (Nakajima et al., 2015). Nevertheless, current research predominantly employs single-species planktonic models, neglecting biofilm-mediated pathogenicity.

This study pioneers a Tri-Species periodontal biofilm model (red/orange/yellow complex representatives) to investigate structured microbial community impacts on: (1) Gut microbiota compositional/functional shifts; (2). Intestinal barrier integrity; (3). Systemic immune activation. Our findings advance understanding of oral-gut axis mechanisms in periodontitis-IBD comorbidity, bridging a critical gap between microbial ecology and clinical pathophysiology.

## 2 Materials and methods

### 2.1 Microbial cultivation, revival and identification

#### 2.1.1 Cultivation and revival of *F. nucleatum*, *P. gingivali*s, and *S. sanguis*

Strains of *Fusobacterium nucleatum* (ATCC 25586), Porphyromonas gingivalis (ATCC 33277), and *Streptococcus sanguinis* (ATCC 10556) were obtained from Beina Biotechnology Co., Ltd. The experimental protocols strictly adhered to established guidelines for anaerobic microbial cultivation. For revival and primary culture, lyophilized bacterial powder was reconstituted with sterile deionized water and subsequently inoculated onto pre-reduced Brain Heart Infusion (BHI) solid medium, which contained 5% defibrinated sheep blood and 0.001% vitamin K1. Cultivation was performed in a tri-gas incubation system (80% N_2_, 10% H_2_, 10% CO_2_) at 37°C, with incubation durations of 5 to 7 days for both *F. nucleatum* and *P. gingivalis*, and 2 to 3 days for *S. sanguinis*. Following successful revival, single colonies of *S. sanguinis* were transferred to BHI liquid medium for 24 hours, while single colonies of *F. nucleatum* and *P. gingivalis* were cultured in BHI liquid medium for 48 hours. The resulting activated bacterial solutions, after two activation cycles, were utilized for subsequent experimental procedures.

#### 2.1.2 Identification of *F.nucleatum, P.gingivalis* and *S.sanguis*

The Gram staining procedure was carried out as follows:

Add a drop of sterile saline to the center of the slide. Using a sterile inoculating loop, scrape a single colony and mix it thoroughly with the saline, spreading it into a thin film. Fix the slide by passing it through the flame of an alcohol lamp. Apply crystal violet stain for 1 minute, then rinse with water to remove the stain. Next, add iodine solution for 1 minute, followed by a rinse with water to eliminate the iodine. Apply decolorizing solution while gently shaking the slide until the purple color disappears, which should take approximately 30 to 60 seconds. Rinse with water to remove the decolorizing solution. Then, add safranin stain for 1 minute, rinse with water to remove the stain, and allow the slide to air dry. Finally, add a drop of cedar oil and observe the color and morphology of the bacteria under the oil immersion lens.

### 2.2 Preparation and measurement of the tri-species biofilm

#### 2.2.1 *In vitro* preparation of a tri-species periodontal biofilm model

Bacteria in the logarithmic growth phase were collected via centrifugation (3,500 × g for 5 minutes at 4°C), resuspended in phosphate-buffered saline (PBS, pH 7.4), and the optical density at 600 nm (OD600) was measured using a UV-visible spectrophotometer (Thermo NanoDrop 2000) and a microplate spectrophotometer (BioTek Synergy H1). The concentrations of the bacterial suspensions were standardized to 1 × 10^9^ CFU/mL for *F.nucleatum* and *P.gingivalis*, and 1 × 10^8^ CFU/mL for *S. sanguinis*. Hydroxyapatite (HA) disks, sterilized using high-temperature and high-pressure methods, were immersed in artificial saliva and pre-treated at 37°C for 4 hours to facilitate the formation of a saliva film on the disks. The HA disks, now coated with the saliva film, were placed at the bottom of a 6-well plate, and bacterial suspensions of *F. nucleatum, P. gingivalis*, and *S. sanguinis* were inoculated onto the HA disks in a concentration ratio of 1:1:1 (v/v). The cultures were then incubated anaerobically at 37°C for 24 hours, with three parallel experiments conducted for each group.

#### 2.2.2 Measurement of the morphological structure of the tri-species biofilm

The morphological characteristics of the biofilm were evaluated using scanning electron microscopy (SEM). The hydroxyapatite (HA) disks with adhered biofilm were gently rinsed with sterile phosphate-buffered saline (PBS) to remove non-adherent bacteria. The disks were then fixed overnight at 4°C in a 2.5% glutaraldehyde solution, followed by dehydration, drying, and coating for electron microscopy. The morphological structure of the biofilm was subsequently examined using SEM at magnifications of 3,500, 12,000, and 15,000 times.

### 2.3 The influence of periodontal pathogens and tri-species biofilm on gut microbiota and intestinal tissue architecture in murine models

#### 2.3.1 Development of animal models

Seven-week-old male C57BL/6 mice, classified as SPF-grade and weighing between 20 and 25 grams, were housed in an independent ventilation system (IVC) under controlled conditions (temperature: 24 ± 1°C, relative humidity: 50 ± 5%, 12-hour light-dark cycle), with unrestricted access to sterilized food and water. All experimental protocols involving animals received approval from the Ethics Committee for Experimental Animals at Zunyi Medical University. The experimental groups consisted of ten mice each: F group (100 μL bacterial suspension containing 1 × 10^9^ CFU of *Fusobacterium nucleatum*, P group (100 μL bacterial suspension containing 1 × 10^9^ CFU of *Porphyromonas gingivalis*, S group (100 μL bacterial suspension containing 1 × 10^8^ CFU of *Streptococcus sanguinis*, and BF group (100 μL suspension containing 1 × 10^9^ CFU of a tri-species biofilm in a 1:1:1 ratio). The control group (H group, n = 10) received an equivalent volume of sterile PBS. Mice were subjected to gavage every other day using a 22G stainless steel gavage needle for a duration of six weeks. Body weight and food consumption were recorded weekly, and at the conclusion of the experiment, colon tissue and fecal samples were collected for further analysis.

#### 2.3.2 Monitoring mouse body weight

At the conclusion of the experimental period (week 6), the mice were continuously observed, and body weight measurements were taken after a 6-hour fasting period, during which they had free access to water. Each mouse was weighed three times, and the average weight was calculated. The data are presented as a percentage change in body weight.

#### 2.3.3 Tissue sample fixation, dehydration, embedding and sectioning

The initial 1 cm segment of colon tissue was excised and fixed in 4% paraformaldehyde for 24 hours, followed by thorough washing with running water overnight. Tissue dehydration was performed using a dehydration machine with a gradient of alcohol, followed by xylene clearing and overnight embedding in paraffin. The samples were embedded using a paraffin embedding machine, and the paraffin blocks were solidified in a cryostat. Subsequently, the paraffin samples were sectioned into 3-4 μm slices using a microtome and stored in slide boxes for future analysis.

#### 2.3.4 Hematoxylin and eosin staining

The sections were placed in a drying oven at 60°C for two hours, followed by two rounds of xylene deparaffinization, each lasting 10 minutes. Rehydration was achieved through a gradient of alcohol, immersing the sections in 100%, 95%, 80%, and 70% alcohol, as well as deionized water, for 2 minutes each. Hematoxylin was applied for 5 minutes, followed by a wash with running water for 1 minute, and immersion in a differentiation solution for 10 to 20 seconds, followed by another wash. Eosin was applied for 3 minutes, and the sections were washed twice with deionized water, each for 1 minute. The sections were then dehydrated in 80%, 95%, and 100% alcohol for 2 minutes each. Xylene clearing was performed twice, each for 5 minutes. Finally, the sections were mounted with neutral gum and coverslips, and subsequently baked in a 60°C oven for 2 hours.

#### 2.3.5 Immunohistochemical staining

The slicing and dehydration steps are the same as in 1.2.6. It is important to choose specially treated slides to prevent detachment during the immunohistochemistry process. Soak in 3% H_2_O_2_ at room temperature for 10 minutes, then wash with distilled water twice. Immerse the slices in 0.01 M sodium citrate buffer, heat in a microwave until boiling, then stop, and repeat this process 2–3 times with 10-minute intervals. Cool at room temperature for 30 minutes, then wash with PBS 1–2 times. After adding the blocking solution, place the slices at room temperature for 20 minutes, then remove excess liquid without washing. Add the primary antibody Anti-Occludin (1:1000) overnight, and for the negative control, add PBS, then wash 3 times for 2 minutes each. Add Bio-goat anti-rabbit IgG and incubate in a 37°C incubator for 30 minutes. Wash with PBS 3 times for 2 minutes each. Add streptavidin-POD working solution and incubate in a 37°C incubator for 30 minutes. Wash with PBS 4 times for 5 minutes each. After adding the chromogenic agent, rinse, counterstain, cover the slides, and observe. Use ImageJ software to analyze the average optical density values of positive signals in the immunohistochemistry results.

#### 2.3.6 RNA extraction

A 4 mm segment of colon tissue was isolated, rapidly frozen in liquid nitrogen, and stored at -80°C for future use. A 100 mg sample of colon tissue was homogenized, and RNA extraction was performed using an RNA extraction kit (Solarbio- Total RNA Extraction Kit, Solaibo Technology Co., Ltd). To the homogenate, 1 mL of lysis buffer was added, mixed vigorously, and allowed to sit at room temperature for 5 minutes. Subsequently, 0.2 mL of chloroform was added, mixed vigorously for 15 seconds, allowed to sit at room temperature for 5 minutes, and then centrifuged at 4°C at 12,000 rpm for 10 minutes. The supernatant was transferred to a new tube, and 500 μL of wash buffer was added to the adsorption column. This mixture was allowed to sit at room temperature for 2 minutes, after which the waste liquid was discarded. The collected supernatant from the previous step was mixed with 200 μL of absolute ethanol, added to the washed adsorption column, allowed to sit for 2 minutes, and the waste liquid was discarded. A total of 600 μL of wash solution (freshly prepared 75% ethanol) was added to the adsorption column, the waste liquid was discarded, and this step was repeated twice. The column was centrifuged at 4°C at 12,000 rpm for 2 minutes, the collection tube was discarded, and the adsorption column was left at room temperature for a few minutes to dry. Finally, 70 μL of RNase-free ddH2O was added to the center of the adsorption column membrane, allowed to sit at room temperature for 5 minutes, and then centrifuged at 12,000 rpm at room temperature for 2 minutes to obtain RNA, which was subsequently quantified for concentration and purity.

#### 2.3.7 Reverse transcription

The reverse transcription process was conducted according to the instructions provided by the reverse transcription kit (PrimeScript™ RT reagent Kit with gDNA Eraser (Perfect Real Time), Takara). In an EP tube, 1 μg of RNA, 2 μL of gDNA Eraser Buffer, 1 μL of gDNA Erase, and enzyme-free water were combined to achieve a total volume of 10 μL. The reaction conditions were set at 42°C for 2 minutes, followed by cooling to 4°C. Subsequently, 4 μL of 5X PrimeScript Buffer 2, 1 μL each of PrimeScript RT Enzyme Mix I and RT Primer Mix, and 4 μL of enzyme-free water were added to the EP tube. The reaction conditions were maintained at 37°C for 15 minutes, followed by a brief incubation at 85°C for 5 seconds, and concluded at 4°C. The resulting cDNA was stored at -20°C.

#### 2.3.8 Quantitative real-time polymerase chain reaction

qPCR reagents were prepared in an eight-tube strip according to the kit instructions (TB Green^®^ Premix Ex Taq™ II (Tli RNaseH Plus), Takara). Each well received 12.5 μL of TB Green, 1 μL of both upstream and downstream primers, 3 μL of cDNA, and 8.5 μL of enzyme-free water. The PCR program was as follows: Step 1: 95°C for 30 seconds; Step 2: PCR reaction at 95°C for 5 seconds, followed by 60°C for 30 seconds, for a total of 40 cycles; Step 3: 95°C for 10 seconds, 65°C for 5 seconds, and 95°C for 5 seconds (**Table 1**).

**Table 1:** PCR primer sequence.

Primer name	Forward primer sequence (5’-3 ‘)	Reverse primer sequence (5’-3’)
GAPDH	AGGTCGGTGTGAACGGATTTG	TGTAGACCATGTAGTTGAGGTCA
B220	GTTATCCACGCTGCTGCCTCAC	TTGGCTGCTGAATGTCTGAGTGTC
TNF-a	GCTACGACGTGGGCTACAG	CCCTCACACTCAGATCATCTTCT
F4/80	GATCCCAGAGTGTTGATGCAA	TTGTACGTGCAACTCAGGACT
L- 18	CAGGCTGTCTTTTGTCAACGA	GACTCTTGCGTCAACTTCAAGG
NOS2	GTGGACGGGTCGATGTCAC	GTTCTCAGCCCAACAATACAAGA
Arg-1	AGGAGCTGTCATTAGGGACATC	CTCCAAGCCAAAGTCCTTAGAG
IL- 1	GAAGGTCCACGGGAAAGACAC	TTCAGGCAGGCAGTATCACTC
Occludin	TTGAAAGTCCACCTCCTTACAGA	CCGGATAAAAAGAGTACGCTGG

#### 2.3.9 Detection of 16S rRNA

The 16S rRNA gene was utilized to evaluate the diversity of gut microbiota. Following the final gavage, fecal samples were systematically collected at a designated time each day, specifically between 11:00 AM and 1:00 PM. Fresh fecal specimens were promptly frozen in liquid nitrogen for five minutes and subsequently stored at -80°C. The amplified region corresponds to the V3-V4 segment of the 16S rRNA gene, utilizing the primers 341F (5’-CCTAYGGGRBGCASCAG-3’) and 806R (5’-GGACTACNNGGGTATCTAAT-3’). Subsequent sequencing and synthesis analyses were performed on the Illumina HiSeq platform (Beijing Novogene Bioinformatics Technology Co., Ltd.). A total of 31 samples underwent 16S rRNA gene sequencing and analysis.

#### 2.3.10 Statistical analysis

Experimental data were analyzed using SPSS 27.0 and GraphPad Prism 10.3, with results presented as means ± standard deviation (SD). A one-way ANOVA was used for comparative analysis among multiple groups, followed by Tukey’s multiple comparison test for exploratory analysis. *P* < 0.05 was considered statistically significant.

## 3 Results

### 3.1 Bacterial culture and detection outcomes of the tri-species biofilm

**Figure 1:**
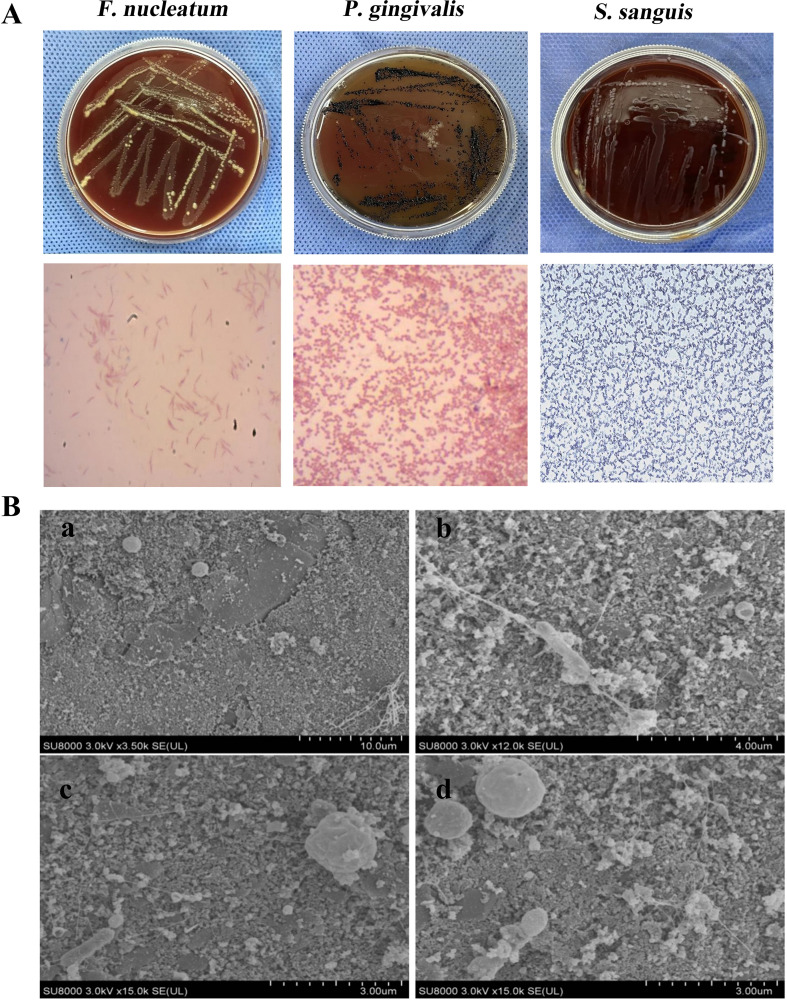
Cultivation of *F.n, P.g, S.s* and Tri-Species biofilm. **(A)** Colony morphology and Gram staining of *F.nucleatum*, *P.gingivalis*, and *S.sanguis*. **(B)** Scanning electron microscopy images of the biofilm: a (×3500), b (×12000), c, d (×15000). Observed were long, thin bacilli and cocci of various sizes, accompanied by an irregularly shaped extracellular polysaccharide matrix that formed a clustered network distribution.

As illustrated in **Figure 1A**, the characteristics of the bacterial colonies are as follows: *F.nucleatum* exhibits prominent colonies with a central dot-like structure observable under oblique light, accompanied by a distinctive hydrogen sulfide odor. Gram staining reveals a red coloration, indicating its classification as a Gram-negative fusiform bacillus. *P.gingivalis* presents brown-black, round, and prominent colonies, emitting an odor reminiscent of indole metabolic products; Gram staining also shows red, confirming its status as a Gram-negative bacillus. *S.sanguis* is characterized by γ-hemolytic white colonies measuring 0.5-1.0 mm in diameter, featuring a “dew-like” raised surface. Gram staining reveals a blue-purple coloration, indicating that it is a Gram-positive coccus. Scanning electron microscopy (SEM) analysis results (**Figure 1B**) demonstrate that the bacterial morphology is consistent and that the structural integrity is maintained, comprising long and slender bacilli alongside cocci of varying sizes, arranged in a clustered network distribution. Additionally, a substantial amount of amorphous extracellular polysaccharide matrix envelops the bacteria, thereby confirming the *in vitro* formation of the Tri-Species periodontal biofilm.

### 3.2 Periodontal pathogenic bacteria and tri-species biofilm induce histopathological alterations in mice intestinal tissue

**Figure 2:**
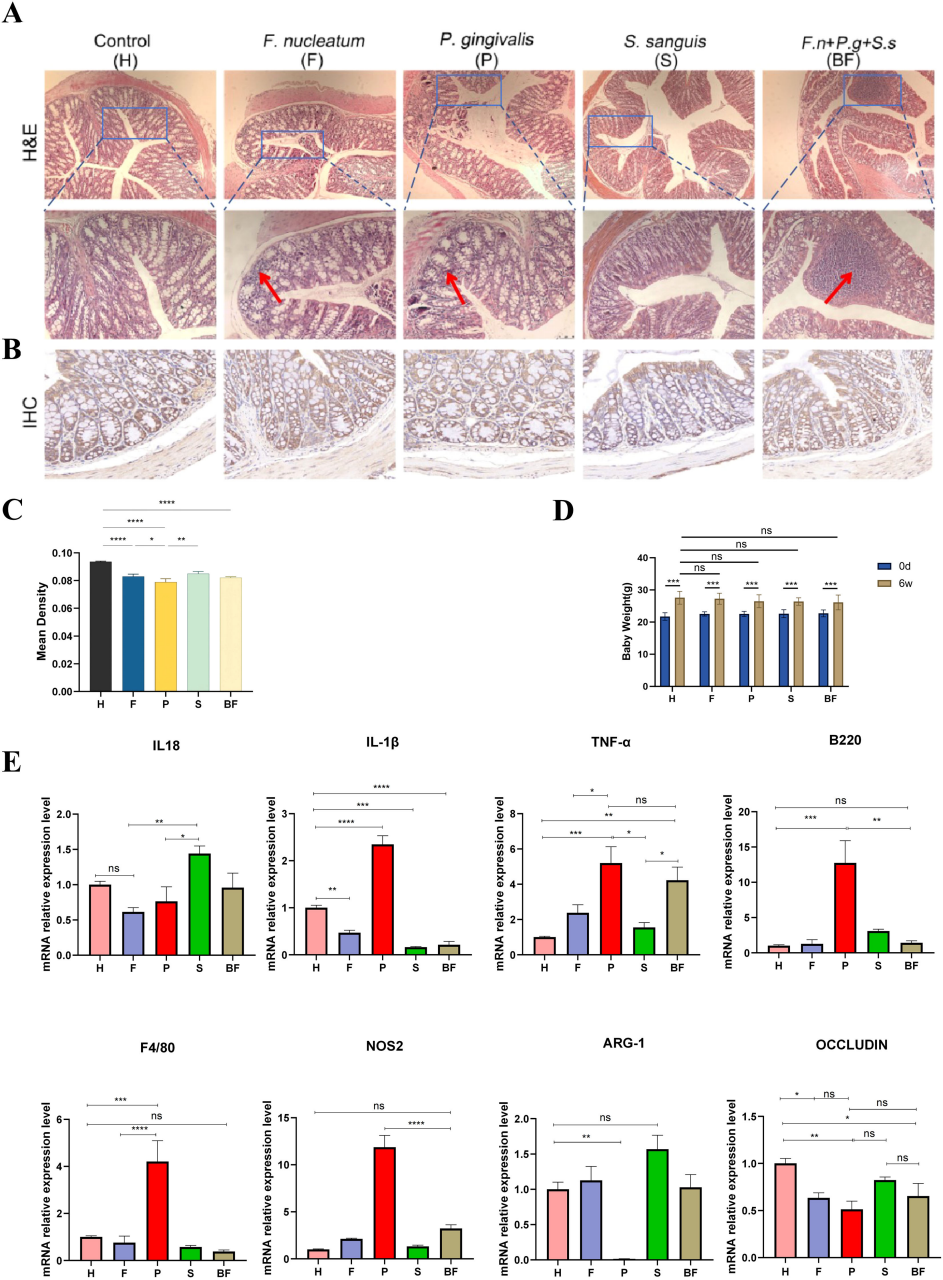
Pathological changes in the colon of mice. **(A)** HE staining of the intestines from five groups of mice, with red arrows indicating inflammatory cell infiltration. **(B)** Immunohistochemical staining of Occludin in the intestines from five groups of mice. **(C)** Average optical density values of Occludin protein from immunohistochemical staining. **(D)** Changes in Mice Body Weight. **(E)** PCR analysis of the colon from five groups of mice, showing the expression levels of IL-18, IL-1β, TNF-α, B220, F4/80, NOS2, ARG-1, and Occludin. (means ± SD, *P < 0.05, **P < 0.01, ***P < 0.001, ****P < 0.0001). ns, no significance.

H&E staining (**Figure 2A**) reveals that the intestinal lumen texture and crypts of mice in the F/P/BF groups exhibit mild deformation, accompanied by a small degree of inflammatory cell infiltration in the submucosa. IHC results (**Figures 2B, C**) indicate a significant increase in the expression of positive cells in the P group (*P* < 0.0001). The bar graph depicting changes in body weight (**Figure 2D**) shows that the body weight of mice across all five groups gradually increases over time. However, the growth rates of the F and P groups are lower than that of the control group, although these differences are not statistically significant (*P* > 0.05). PCR detection (**Figure 2E**) of inflammatory factor gene expression in the colon tissues of mice from each group reveals that IL-1β, TNF-α, B220, F4/80, and NOS2 are significantly increased in the P group (*P* < 0.001), while Arg-1 expression is significantly reduced (*P* < 0.01). In the BF group, a notable increase in TNF-α expression was observed(*P* < 0.01). Additionally, the expression of Occludin is significantly decreased in the F, P, and BF groups, with the most pronounced reduction observed in the P group (*P* < 0.01).

### 3.3 Periodontal pathogenic bacteria and tri-species biofilm induce dysbiosis in the gut microbiota of mice

**Figure 3:**
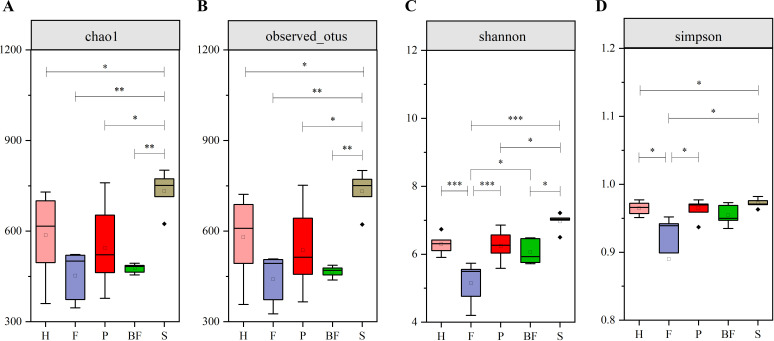
Analysis of α diversity in mice gut microbiota. **(A)** Chao Index. **(B)** OTU Index. **(C)** Shannon Index. **(D)** Simpson Index. These results indicate that periodontal pathogens and the biofilm of three bacterial species contribute to alterations in the richness and diversity of the mouse gut microbiome. (means ± SD, *P < 0.05, **P < 0.01, ***P < 0.001).

**Figure 4:**
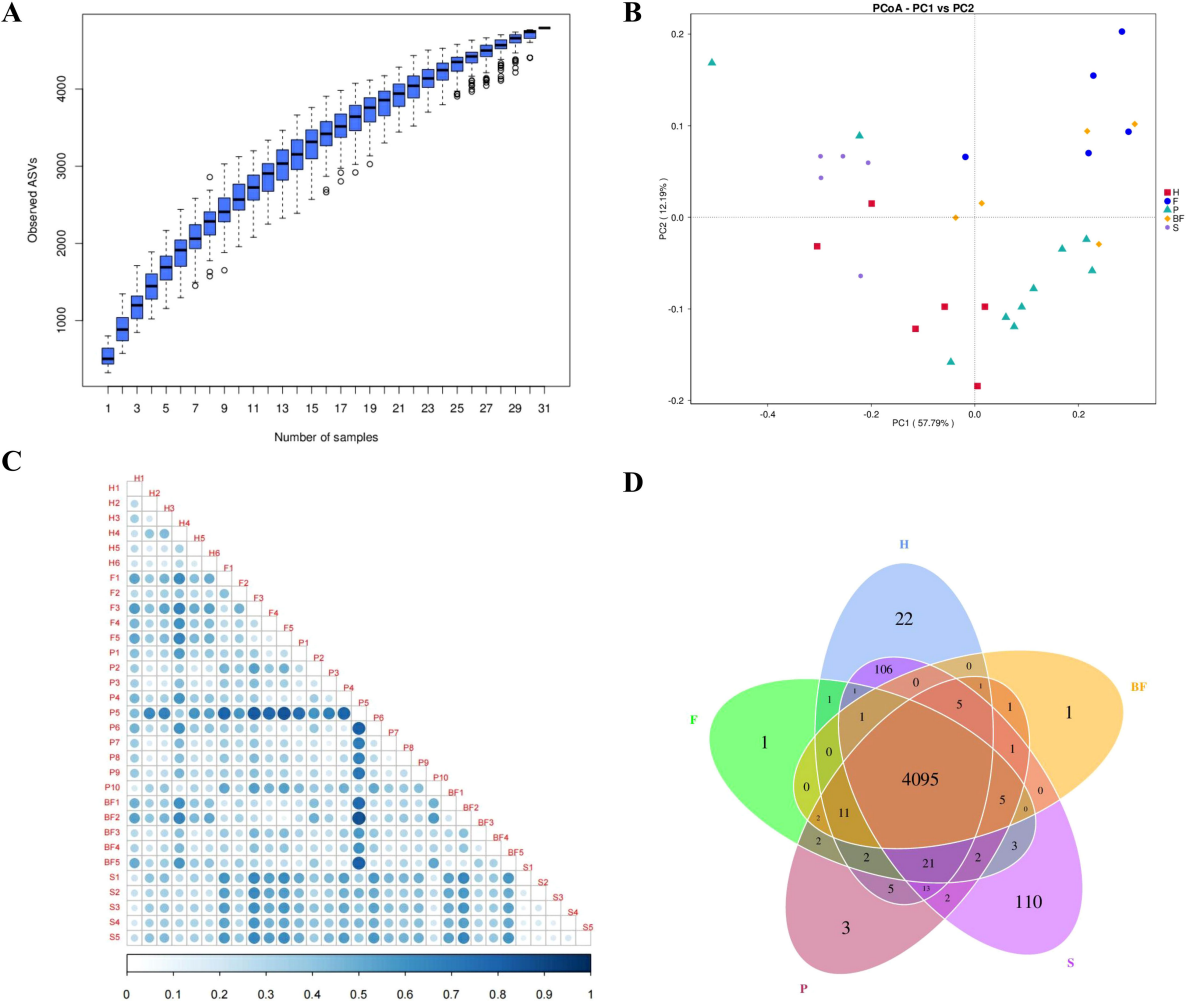
Analysis of β diversity in mice gut microbiota. **(A)** Box plot of species accumulation. **(B)** PCoA analysis. **(C)** Heatmap of the distance matrix. **(D)** ASV Venn Diagram.

**Figure 5:**
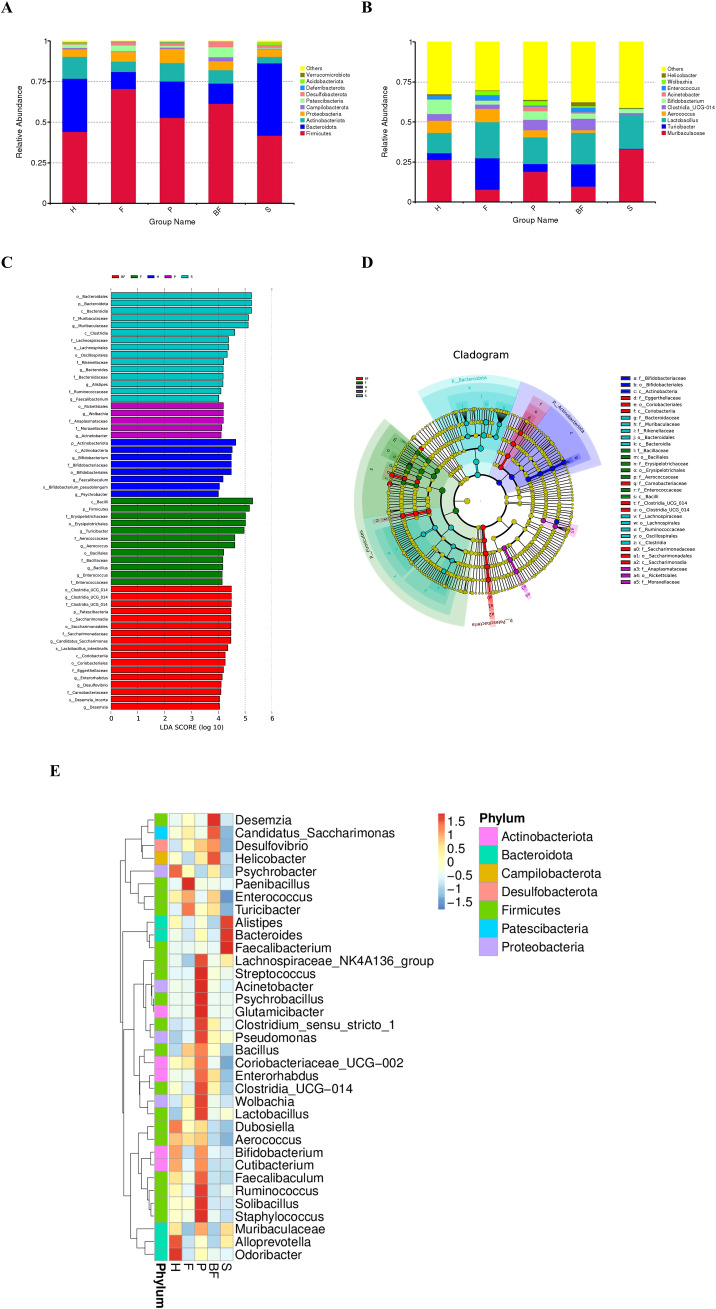
Analysis of gut microbiota in mice. **(A)** Bar chart illustrating species composition at the phylum level. **(B)** Bar chart depicting species composition at the genus level. **(C)** LEfSe analysis LDA bar chart. **(D)** LEfSe analysis taxonomic branch diagram. **(E)** Species abundance clustering diagram.

Following the completion of mouse modeling at week 7, samples were collected. The results obtained from 16S rRNA sequencing indicated that the presence of periodontal pathogenic bacteria and biofilms from three bacterial species contributed to the dysbiosis of the gut microbiota in C57BL/6 mice. In the alpha diversity analysis, the Chao1 and OTU indices for F/P/BF group were lower than those of control group, although there is no statistical significance. Conversely, S group exhibited a significant increase (*P* < 0.05). The Simpson and Shannon indices for F group demonstrated a significant reduction (*P* < 0.05) (**Figures 3A-D**). Cumulative box plots for five groups reached a plateau, suggesting that the sequencing results were sufficient to represent the diversity present in the current samples (**Figure 4A**). In the beta diversity analysis, the gut microbiota of the F/P/S/BF group exhibited significant deviations from the control group, as demonstrated by PCoA and distance matrix heatmap analyses based on UniFrac distances (**Figures 4B, C**). The Venn diagram illustrated the number of shared and unique genes within the gut microbiota across these five groups (**Figure 4D**). Notably, the species composition of the gut microbiota revealed substantial differences (**Figures 5A-E**). At the phylum level, the study identified an increase in the *Firmicutes/Bacteroidetes* (F/B) ratio in F/P/BF group; a significant rise in the abundance of *Proteobacteria* in P group; an increase in *Desulfovibrio* abundance in F/P/BF group; and a decrease in *Verrucomicrobiota* in F/S group, with the representative genus *Akkermansia* closely associated with the integrity of the mucus layer (**Figure 5A**). At the genus level, the study observed a decrease in the abundance of *Muribaculaceae* in F/P/BF group, with a particularly pronounced decline in F/BF group (**Figure 5B**). In contrast, the abundance of *Turicibacter*, a member of the phylum *Firmicutes*, increased, which may influence metabolic functions, with significant increases noted in F/BF group. Additionally, the abundance of Lactobacillus rose in F/P/S/BF group, likely contributing to the regulation of gut pH through lactic acid metabolism. Conversely, the abundance of *Bifidobacterium* and *Parabacteroides* decreased in F/P/BF group, both of which are recognized for their anti-inflammatory properties. Furthermore, the abundance of *Enterococcus* increased in F/BF group, while *Acinetobacter* abundance significantly increased in P group, with both genera classified as opportunistic pathogens (**Figures 5C, D**).

## 4 Discussion

Our study elucidates novel mechanistic pathways through which periodontal pathogens disrupt gut homeostasis via oral-gut axis dysregulation. Cross-population cohort studies have demonstrated that individuals diagnosed with IBD are more susceptible to periodontal disease compared to healthy control populations (Haznedaroglu and Polat, 2023). Notably, patients with IBD exhibit distinct oral health profiles, characterized by a higher frequency of periodontal interventions, such as scaling, compared to the control group, along with lower oral health scores. The observed comorbidity between periodontitis and IBD may stem from bidirectional microbial-immune crosstalk (Kato et al., 2020), wherein the daily ingestion of 1.5×10^12 oral bacteria through salivary flow (600 swallows/day) creates persistent gut colonization pressure (Chen et al., 2010). While gastric acid eliminates >99% of planktonic *P. gingivalis*, biofilm encapsulation enhances acid resistance (50% survival at pH ≤ 3 *vs* 1% in planktonic state), enabling pathogenic translocation (Sato et al., 2017). The tri-species biofilm model (*F.n, P.g*, *S.s*) recapitulated key ecological features of subgingival plaque, including EPS-mediated structural integrity (SEM-confirmed) and acid tolerance. Notably, biofilm administration induced differential immune activation.

Research indicates that the Occludin protein, located on the surface of cell membranes, plays a crucial role in tightly linking adjacent epithelial cells, thereby providing resistance against the invasion of exogenous substances. A reduction in the expression of the Occludin protein may serve as an indicator of compromised intestinal barrier function (Kuo et al., 2022). This finding aligns with the observed decrease in Occludin protein levels in the colon tissues of F/P/BF group in the present study. Furthermore, when intestinal barrier integrity is compromised, the permeability of the mucosal epithelium is enhanced, facilitating the translocation of bacteria and triggering the production of inflammatory cytokines (Botía-Sánchez et al., 2023). This investigation employed quantitative reverse transcription qRT-PCR to demonstrate that F group induces an upregulation of TNF-α expression in colon tissue. In contrast, P group elicits a more extensive pro-inflammatory response, characterized by significant increases in IL-1β, TNF-α, B220, F4/80, and the M1 marker NOS2, while the expression of the M2 marker Arg-1 is markedly diminished. We hypothesize that this phenomenon may be associated with *P.g*, a well-established periodontal pathogen that plays a “Keystone” role; its LPS can activate NF-κB via the TLR4/MyD88 signaling pathway, thereby promoting the synthesis of IL-1β and TNF-α. Furthermore, its distinctive gingipains can cleave intercellular adhesion proteins, such as E-cadherin, facilitating bacterial invasion of the epithelium and activating the NLRP3 inflammasome (Hajishengallis, 2014; Jia et al., 2019). This mechanism may elucidate the increased expression of IL-1β and the decreased expression of Occludin protein observed in the P group. The presence of plaque biofilms enhances the bacteria’s capacity to withstand various external stimuli, thereby exerting pathogenic effects. Consequently, we propose that periodontal plaque biofilms, particularly the non-adherent subgingival biofilms, are more likely to colonize the intestines via the oral route through the gastric environment, as opposed to planktonic bacteria, thus influencing the micro-ecological structure of the gut microbiota. However, our findings indicate that intervention with the tri-species biofilm (*F.n* + *P.g* + *S.s*, BF group) resulted in a significant elevation of TNF-α compared to control group, while the expressions of IL-1β, B220, and F4/80 were significantly lower than those in the P group(*P* < 0.001). We speculate that these observations may be attributed to the ecological interactions among the tri-species biofilm. Research has demonstrated that hydrogen peroxide and blood chain factors produced by *S.s* can inhibit the growth of *P.g* and *F.n*, thereby reducing the adhesion capacity and pathogenicity of these bacteria (Diaz, 2012; da Costa Rosa et al., 2021). Additionally, *F.n* interacts with the immune cell inhibitory receptor TIGIT via the fusobacterium protein Fap2, which protects it from natural killer (NK) cell-mediated cytotoxicity and immune cell attacks, thereby mitigating excessive inflammatory responses (Gur et al., 2015). Furthermore, competition for nutrients within the microbial community of the biofilm may limit the proliferation of *P.g* and decrease the release of its pathogen-associated molecular patterns (PAMPs). Notably, our findings indicate that NOS2 (M1) expression was elevated in the biofilm group, while Arg-1 (M2) expression was not suppressed. This suggests that the microbial community within the plaque biofilm may activate mixed macrophage (M1/M2) phenotypes through the activation of TLR2/TLR6 heterodimers (Kawai et al., 2024). These results imply that oral pathogens can reshape the gut microbiota through various mechanisms, including the modulation of immune responses and metabolic effects, which lead to alterations in gut structure that ultimately affect overall health. The ecological competition and synergistic interactions within the microbial community of the biofilm may influence disease through a dynamic balance of “virulence-symbiosis”.

Previous research has indicated that a reduction in alpha diversity serves as a reliable marker for disease-associated dysbiosis (Arimatsu et al., 2014). The current study observed a decrease in both the abundance and alpha diversity of gut microbiota in mice harboring major periodontal pathogenic bacteria, specifically *F.n* and *P.g*, as well as in a tri-species biofilm group comprising *F.n*, *P.g*, and *S.s*. These findings align with the microbial profiles typically observed in patients with IBD. Notably, the relative abundance of *Firmicutes* was significantly elevated in the groups exposed to *F.n*, *P.g*, and the biofilm, while the abundance of *Bacteroidetes* was diminished, resulting in an increased F/B ratio compared to control group. This alteration in microbial composition suggests a potential commonality with the pathological mechanisms underlying IBD (Jia et al., 2024). Furthermore, Wu et al. demonstrated that *F.n* can ectopically colonize the gastrointestinal tract, thereby modifying the gut microbial structure and inhibiting butyrate production via the AMPK signaling pathway, which may facilitate the progression of colorectal cancer (Wu et al., 2024). As the severity of the disease escalates, a corresponding decrease in *Bacteroidetes* and an increase in *Firmicutes* within the gut microbiota have been observed. Additionally, in various systemic inflammatory conditions, an increase in the *Firmicutes* to *Bacteroidetes* ratio has been documented (Ley et al., 2005), corroborating the findings of elevated *Firmicutes* and reduced *Bacteroidetes* in F/P/BF group in the present study.

The observed enrichment of *Proteobacteria* in the intestines of P group mice demonstrates a strong positive correlation with previously identified markers of intestinal barrier damage. *Proteobacteria* are known to activate inflammatory pathways through the secretion of LPS, which can induce apoptosis in intestinal epithelial cells and promote the release of TNF-α (Sun et al., 2021). This suggests that the presence of *Proteobacteria* may exacerbate intestinal damage in conjunction with pathogenic bacteria. *Muribaculaceae*, a bacterial family within the Bacteroidetes phylum, plays a crucial role in the production of short-chain fatty acids from both endogenous sources (mucin polysaccharides) and exogenous sources (dietary fiber). Our findings indicate a decrease in the abundance of *Muribaculaceae* in the F/P/BF group, which may lead to a reduction in butyrate concentration within the intestine. This reduction could adversely affect the intestinal acidic environment, facilitating the colonization of pathogenic bacteria and further compromising intestinal barrier function (Zhu et al., 2024b). Furthermore, *Muribaculaceae* has a cross-feeding relationship with beneficial probiotics, such as *Bifidobacterium* and *Lactobacillus*. In this study, we observed a decline in the anti-inflammatory probiotics *Bifidobacterium* and *Parabacteroides* in F/P/BF group, while the opportunistic pathogen *Enterococcus* exhibited significant enrichment in F/BF group, and *Acinetobacter* showed increased abundance in P group. Numerous studies have established that *Lactobacillus* and *Bifidobacterium* are essential for maintaining the integrity of the intestinal barrier, promoting immune tolerance, and reducing bacterial translocation across the intestinal mucosa (Rubin et al., 2022; Zhao et al., 2024). Notably, members of the phylum Firmicutes, particularly the genus *Turicibacter*, were found to be elevated in the F and BF groups. This genus has the ability to modulate host genes associated with bile acid and lipid metabolism, leading to reductions in serum cholesterol, triglycerides, and adipose tissue mass, thereby functioning as a regulator of host fat biology (Lynch et al., 2023). Additionally, we observed an increase in *Desulfovibrio* in the F/P/BF group. These anaerobic bacteria reduce sulfate to produce hydrogen sulfide (H_2_S), which has a toxic effect on intestinal epithelial cells, increasing intestinal sensitivity and resulting in symptoms such as abdominal pain and bloating (Verstreken et al., 2012). Clinical studies have confirmed that an increase in *Desulfovibrio* is a significant characteristic of intestinal polyps and ulcerative colitis (UC).

The findings presented suggest that periodontal pathogens contribute to dysbiosis within the gut microbiota through ectopic colonization, compromising the integrity of intestinal epithelial tight junctions. This process subsequently triggers immune cell-mediated inflammatory responses that negatively impact gut health. Such insights provide a theoretical framework for understanding the role of the oral-gut axis in the pathogenesis of IBD. However, it is important to acknowledge that the simplified three-species model employed in this study represents only 0.03% of the subgingival microbial species. While this model can mimic certain ecological characteristics, it fails to accurately replicate the complex network of interspecies interactions found in a natural biofilm. Furthermore, the acute exposure protocol utilized in the gavage model may not adequately reflect the chronic effects of microbial invasion experienced by patients with clinical periodontal disease, which can persist for extended periods. Consequently, future investigations should incorporate metagenomics and chip technology to facilitate dynamic analyses.

## Data Availability

The datasets presented in this study can be found in online repositories. The names of the repository/repositories and accession number(s) can be found in the article/**Supplementary Material**.

## Supplementary material

Supplementary Figure 1 Analysis of species differences among T-test groups at the phylum level. **(A)** H-F. **(B)** H-S. **(C)** H-BF.

Supplementary Figure 2 Analysis of species differences among T-test groups at genus level. **(A)** H-F. **(B)** H-P. **(C)** H-S. **(D)** H-BF.
