# Supplementary figures and images for: Effects of oral gavage with periodontal pathogens and plaque biofilm on gut microbiota ecology and intestinal tissue architecture in mice: a mechanistic study
Source: Front Cell Infect Microbiol. 2025 Aug 8;15:1589055. doi: 10.3389/fcimb.2025.1589055 (PMC12370644; doi:10.3389/fcimb.2025.1589055)

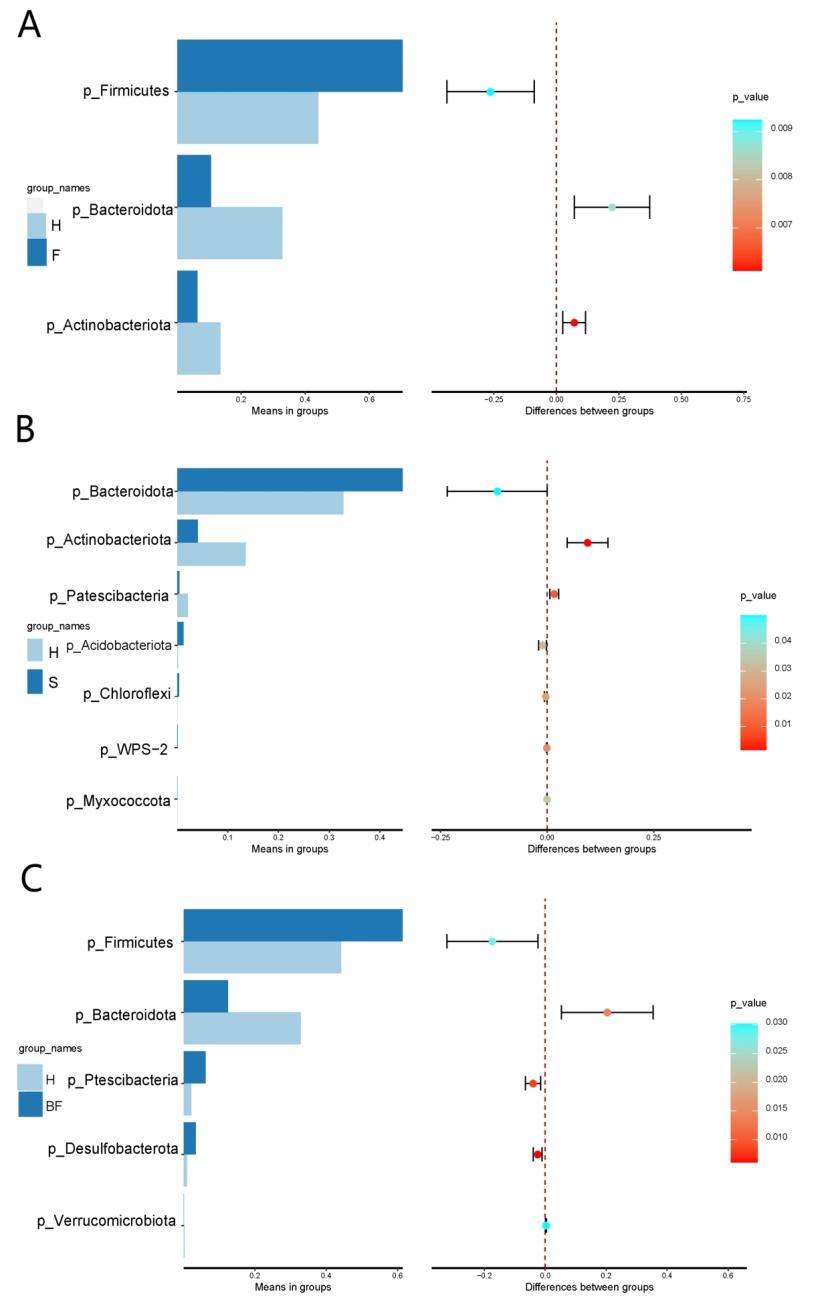

Supplement: Supplementary Figure 1 — Analysis of species differences among T-test groups at the phylum level. (A) H-F. (B) H-S. (C) H-BF. [file Image1.jpg]

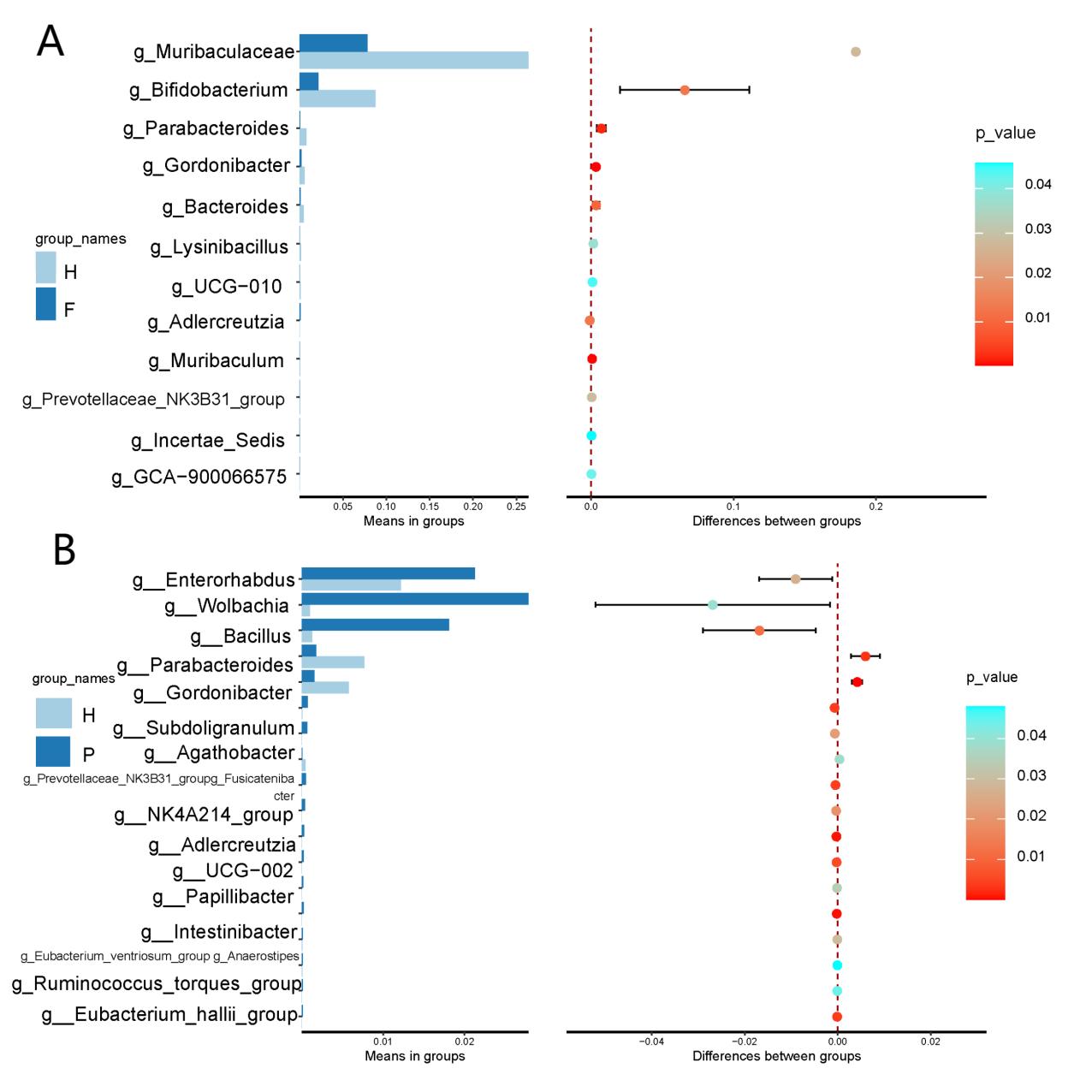

Supplement: Supplementary Figure 2 — Analysis of species differences among T-test groups at genus level. (A) H-F. (B) H-P. (C) H-S. (D) H-BF. [file Image2.jpg]

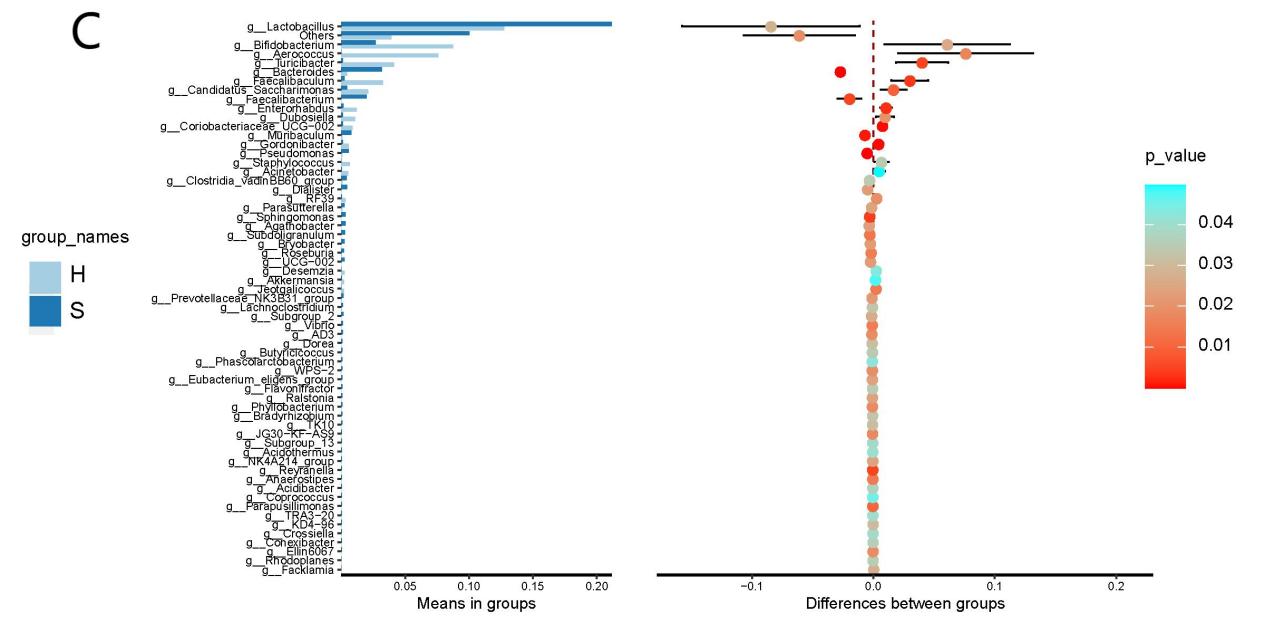

Supplement: Supplementary file 3 [file Image3.jpg]

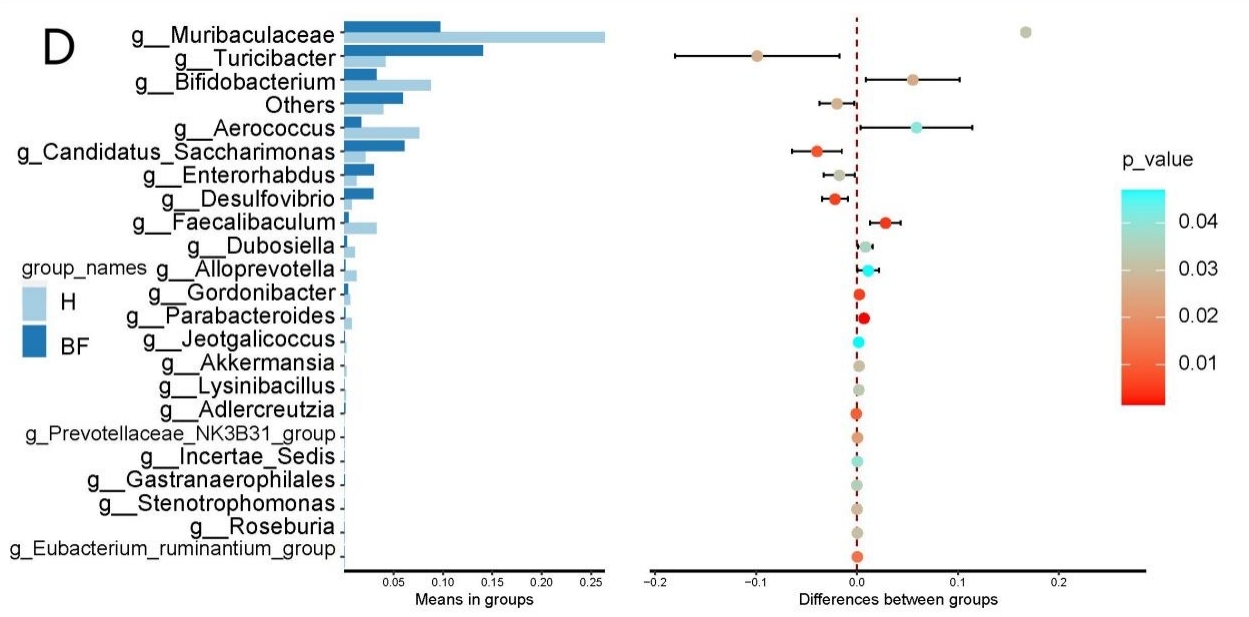

Supplement: Supplementary file 4 [file Image4.jpg]
